# Influence of Age and Body Weight on Supramammary Lymph Node Morphology and Weight in Holstein Dairy Cows

**DOI:** 10.3390/vetsci13060525

**Published:** 2026-05-28

**Authors:** Ran Guan, Chaoyun Yang, Zhiqiang Hu, Songhua Hu

**Affiliations:** 1 Key Laboratory of Animal Epidemic Disease Detection and Prevention in Panxi District, College of Animal Science, Xichang University, Xichang 615013, China; 2 Department of Veterinary Medicine, College of Animal Sciences, Zhejiang University, Hangzhou 310058, China

**Keywords:** supramammary lymph nodes, Holstein dairy cows, udder health, mastitis, immune response

## Abstract

This study aimed to investigate the morphological characteristics and weight of supramammary lymph nodes (SMLNs) in Holstein dairy cows and to evaluate the effects of age and body weight (BW) on these parameters. A total of 19 cows (aged 2–9 years) culled due to reproductive failure (including repeated insemination failure, confirmed open status, or abortion) were divided into 3 groups: high-age (H, ≥6 years, *n* = 6), middle-age (M, 3–5 years, *n* = 6), and low-age (L, 2 years, *n* = 7). Results demonstrated that the weight of SMLNs generally increased with age, although lower values were observed in the limited number of cows older than 7 years. The length and width of SMLNs observed here were markedly larger than those reported in previous ultrasonographic studies. The H group had significantly heavier left, right, and bilateral SMLNs compared to the L group (*p* < 0.01). The M group exhibited significantly higher BW than the L group (*p* < 0.01). Correlation analysis revealed a significant positive association between BW and right SMLN weight in the L group (r = 0.774, *p* < 0.05), and a strong bilateral concordance in SMLN weight across all cows (r = 0.859, *p* < 0.001). These preliminary findings suggest that SMLN morphology and weight are associated with age and BW, although larger prospective studies with complete health and lactation records are needed to confirm these relationships.

## Simple Summary

The supramammary lymph nodes (SMLN), located just above a cow’s udder, play a critical role in immune surveillance and defense against mammary infections such as mastitis, yet their normal size, number, and weight vary greatly among individual cows. By examining 19 culled Holstein dairy cows of different ages (2–9 years) from an on-farm slaughterhouse due to reproductive failure, we found that older cows (6+ years) had SMLNs roughly three times heavier than those in young cows (2 years; all primiparous or nulliparous heifers), although a decline was observed in cows older than 7 years. Heavier cows also tended to have heavier SMLNs, especially in middle-aged cows (3–5 years). While the weights of left and right SMLNs were nearly always similar, the number on each side differed surprisingly—ranging from just one to as many as five. These natural variations help veterinarians better recognize abnormal SMLNs during health checks. They also highlight why treatments or vaccines injected near these nodes should consider individual differences in location and size. These baseline data may help veterinarians interpret SMLN enlargement observed during clinical palpation or ultrasonographic examination. Ultimately, understanding the morphological diversity of SMLN may support healthier cows and safer milk production for everyone.

## 1. Introduction

The supramammary lymph nodes (SMLNs) in dairy cows are critical for immune surveillance and disease resistance, particularly in combating mastitis. Located on the dorso-caudal surface or superficially in the inguinal region near the base of the udder, these nodes are responsible for draining lymphatic fluid from the mammary gland. This function is essential for immune surveillance, pathogen filtration, and immune cell activation [1]. The mammary gland itself is a complex integumentary structure. In cattle, it is located in the inguinal region and comprises four individual glands (quarters) grouped into the udder, which is supported by an elaborate suspensory system capable of bearing over 50 kg of tissue during peak lactation [2]. The lymphatic drainage of the udder converges predominantly at the SMLNs, which vary in number from 1 to 7 and measure 4–10 cm in size, located above the caudal border of the mammary gland base [2]. Comparable anatomical principles have been described in other mammalian species. For example, recent studies of human breast anatomy have highlighted that the mammary glandular tissue is organized within a membrane-bound central structure (the corpus mammae), enveloped by fascial layers that also contribute to lymphatic and structural organization [3]. This cross-species perspective reinforces the notion that precise anatomical knowledge of the mammary gland and its associated lymphoid structures is foundational for understanding immune defense mechanisms.

Studies have highlighted the importance of SMLNs in optimizing immune responses through targeted vaccinations and immunomodulators. Mastitis, caused by a wide range of bacterial pathogens including *Staphylococcus aureus* (*S. aureus*), *Streptococcus* spp., and coliforms, remains one of the most economically devastating diseases in dairy production worldwide, associated with reduced milk yield, deteriorated milk quality, and elevated treatment costs [4,5]. For instance, Guidry et al. [6] demonstrated that injecting a *S. aureus* vaccine near the SMLNs in Jersey cows induced a robust immune response, enhancing the phagocytic ability of dry cow secretions and colostrum. Similarly, Guan et al. [7] found that combining *S. aureus* bacterin inoculation with intramammary infusion of nisin Z significantly reduced intramammary infections and improved milk quality. These studies underscore the anatomical and functional significance of SMLNs in localized immunity and udder health.

Understanding the morphology, quantity, and weight of these lymph nodes is crucial for diagnosing udder infections and systemic diseases. Moreover, individual variations in SMLNs could influence the efficacy of immunomodulators and vaccines. Although early anatomical descriptions of bovine SMLNs were reported more than a century ago, contemporary direct morphometric data from Holstein dairy cows remain limited. Most prior reports relied on ultrasonographic measurements in live animals, which inherently limit accuracy. Indeed, ultrasonographic studies in small ruminants, such as a recent investigation of Saanen goats, demonstrated that the SMLNs appear as hypoechoic, oval, well-demarcated structures on ultrasound, with node dimensions positively correlated with age but not reliably predictive of subclinical mastitis status [8]. This suggests that while ultrasonography is a valuable non-invasive tool for SMLN assessment, it has inherent limitations in resolution and measurement accuracy compared with direct anatomical dissection. Indeed, recent advances in bovine udder ultrasonography have demonstrated its utility in detecting structural changes in mammary tissue during clinical and subclinical mastitis, including alterations in glandular cistern dimensions [9]; however, ultrasonographic assessment of lymph node morphology remains constrained by resolution and operator variability, underscoring the added value of direct post mortem dissection for establishing morphometric baselines. Although early anatomical descriptions of bovine SMLNs were reported more than a century ago, contemporary direct morphometric data from modern Holstein dairy cows remain limited. Therefore, this study used post mortem dissection to characterize SMLN number, morphology, dimensions, and weight across age groups.

## 2. Materials and Methods

### 2.1. Sample Collection and Preparation

This study was conducted from July to September 2019 at the Qiaosi dairy farm (Hangzhou, Zhejiang Province, eastern China; 30° N, 120° E), a region characterized by a subtropical monsoon climate with hot, humid summers (mean summer temperature ~35 °C) and mild winters. The farm housed approximately 3000 Holstein dairy cows under standardized management practices, including total mixed ration (TMR) feeding and year-round indoor housing. Individual records, including parity, culling reason, and most recent daily milk yield, were retrieved from the herd management system DairyComp 305 (DC 305, version 2018; Valley Agricultural Software [VAS], Tulare, CA, USA). Detailed characteristics of all 19 cows are provided in Table 1. All cows were culled primarily due to reproductive failure (repeated insemination failure, confirmed open status, or abortion). Body condition score (BCS) was not systematically recorded for all cows in the herd management system and therefore could not be included in this analysis.

Complete lifetime mastitis histories (including subclinical episodes) and vaccination records were not available for all cows, as the system did not retain comprehensive individual health documentation. Therefore, prior subclinical mastitis, chronic immune stimulation, or prior vaccination as contributing factors to SMLN enlargement in certain individuals were not definitively excluded. This represents a recognized limitation of the retrospective study design and should be considered when interpreting inter-individual comparisons, particularly given the small sample size (*n* = 19). All cows were sourced from the farm’s onsite slaughterhouse. The SMLNs were carefully dissected from the dorso-caudal surface of the udder, ensuring minimal damage to the surrounding tissues. Each SMLN was immediately weighed using an electronic scale (YP-R10002, Shanghai Guangzheng Medical Instrument Co., Ltd., Shanghai, China), and its morphology was documented through photographic records.

### 2.2. Morphological Analysis

The identification of each SMLN was confirmed visually based on its anatomical location, characteristic shape (oval to bean-shaped), and surface appearance. The morphology of the SMLNs was assessed based on their shape, color, and surface characteristics. The number, length, and width of SMLNs on each side of the udder were recorded, and any variations between the left and right sides were noted. The color of the SMLNs was described as grayish white, grayish yellow, or congested with blood vessels.

### 2.3. Statistical Analysis

The data on the number, weight, and morphology of the SMLNs were analyzed using descriptive statistics. The dairy cows aged 2–9 years were divided into three groups: high-age group (H, ≥6 years, *n* = 6), middle-age group (M, 3–5 years, *n* = 6), and low-age group (L, 2 years, *n* = 7; consisted of primiparous or nulliparous heifers). All statistical analyses were performed using R software (v4.3.1, https://www.R-project.org/). For ANOVA, normality was checked via the “rstatix” package (v0.7.2, https://CRAN.R-project.org/package=rstatix, accessed on 1 April 2025), and homogeneity of variance was assessed using the “car” package (v3.1-2, https://cran.r-project.org/web/packages/car/index.html, accessed on 1 April 2025). A linear model (y ~ age) of the form *Y*_ij_ = *μ* + *α_i_* + *ε_ij_* was fitted with “rstatix” functions, where *Y*_ij_ denotes the outcome variable (e.g., SMLN weight) for the *j*-th individual in age group *i*; *μ* is the overall intercept (grand mean); *α_i_* represents the fixed effect of age group *i* (L, M, or H); and *ε_ij_* is the residual error term assumed to be independently and normally distributed with mean zero. The model was implemented using the lm() function in R, with F-statistics and *p*-values computed. Group differences were statistically significant (α = 0.05), and Tukey’s HSD post hoc comparisons were done with the “tukey_hsd” function. The significance was marked with asterisks (* *p* < 0.05; ** *p* < 0.01; *** *p* < 0.001). Unlike age, which was used as a categorical grouping variable (L, M, H) for between-group comparisons via one-way ANOVA, BW was treated as a continuous quantitative variable in the correlation analysis. Pearson’s correlation coefficients were calculated between BW and SMLN weight variables both across all cows and within each age group separately, allowing assessment of the linear relationship between BW and SMLN characteristics independent of age stratification. For correlation analysis, Pearson’s correlation coefficient and Bonferroni-corrected *p*-value matrices were calculated using the “Hmisc” package (v4.8-5.0, https://CRAN.R-project.org/package=Hmisc, accessed on 1 April 2025), with results visualized via the “ggcorrplot” packages (https://CRAN.R-project.org/package=ggcorrplot, accessed on 1 April 2025), “ggcor” packages (https://github.com/houyunhuang/ggcor, accessed on 1 April 2025) If the webpage is temporarily inaccessible, the package can be installed directly in R using: devtools::install_github(“houyunhuang/ggcor”), and “ggplot2” packages (https://CRAN.R-project.org/package=ggplot2, accessed on 1 April 2025), showing correlation strength through color gradients and significance with asterisks.

Given the exploratory nature of this study and the limited sample size (*n* = 19, with *n* = 6–7 per age group), a post hoc statistical power analysis was conducted using the “pwr” package (v1.3-0, https://CRAN.R-project.org/package=pwr, accessed on 1 April 2025) in R to characterize the analytical constraints of the primary comparisons. The achieved statistical power was evaluated for three analytical frameworks: (1) one-way ANOVA comparing bilateral SMLN weight across three age groups, (2) pairwise post hoc comparisons using Tukey’s honestly significant difference (HSD) test with family-wise error rate correction, and (3) Pearson correlation analyses within each age group. For the one-way ANOVA, the effect size was quantified using Cohen’s f, calculated from the group means and pooled standard deviation reported in Table 2. For pairwise comparisons, statistical power was estimated based on a medium effect size (Cohen’s d ≈ 0.8) at a significance level of α = 0.05. For within-group correlation analyses (*n* = 6–7 per group), the minimum detectable correlation coefficient was determined at 80% power (α = 0.05, two-tailed). ANCOVA, including BW and lactation stage, was considered but not used as the primary model because lactation stage was unavailable or not applicable for nulliparous animals and was structurally confounded with age group in this small dataset.

## 3. Results

**Figure 1:**
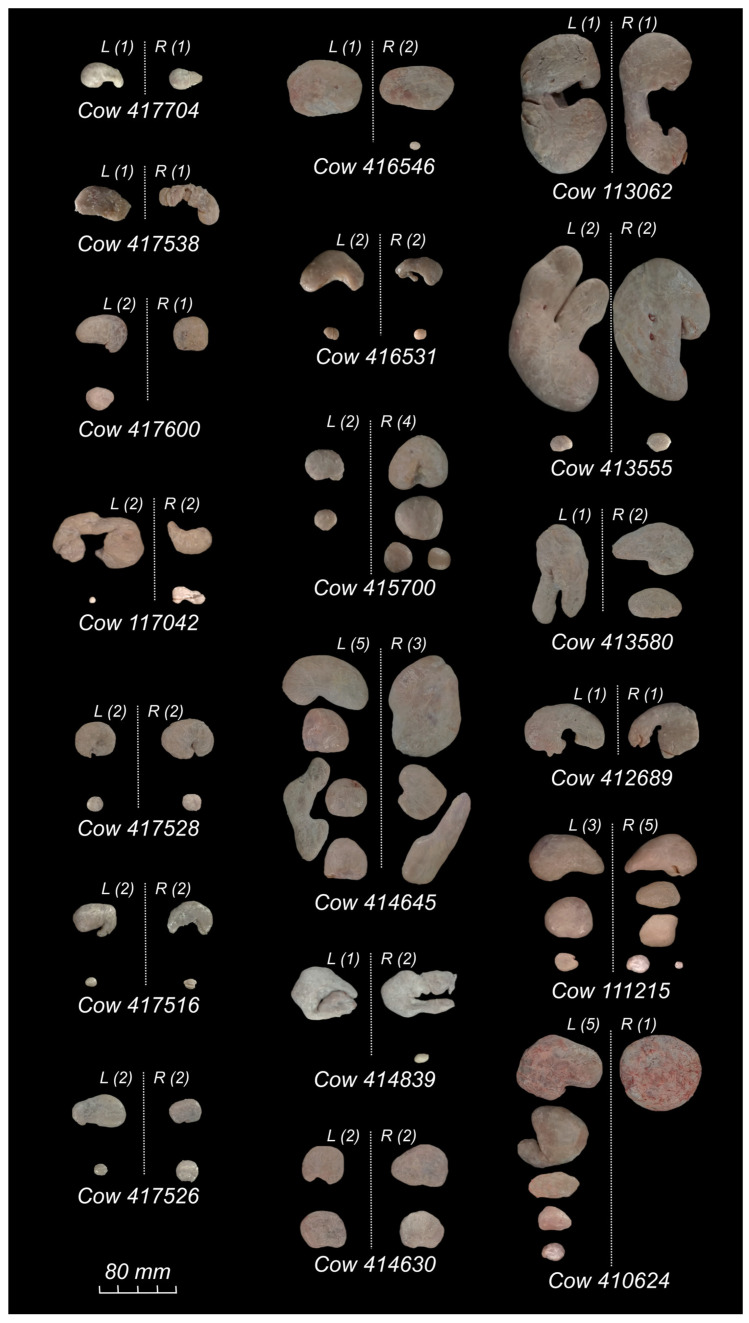
The morphology of SMLNs isolated from the left or right side of the udder in 19 slaughtered Holstein dairy cows. For each cow, the left SMLN(s) are displayed on the left and the right SMLN(s) on the right of each panel.

The age, BW, parity, culling reason, lactation stage, and recent daily milk yield of the 19 Holstein dairy cows are shown in Table 1. There was substantial diversity in the morphology, number, weight, length, and width of SMLNs (Table 2 and Figure 1). Each isolated SMLN was confirmed visually as described in Section 2.2. The SMLNs exhibited a range of colors, from grayish white to grayish yellow, with irregular shapes. One cow had SMLNs that were congested with blood vessels, while the others showed a more uniform appearance. The total number of SMLNs isolated per cow ranged from 2 to 8 (median: 4), with the majority of cows (*n* = 11) yielding bilateral totals of 2 (four cows) or 4 (seven cows) nodes. The remaining eight cows had different numbers of SMLNs on each side. Among these, two cows had up to 5 SMLNs on one side (Cow 414645: L5/R3; Cow 410624: L5/R1), which was the maximum observed in this study. At the individual level, the bilateral SMLN weight ranged from 16.6 g (Group L) to 443.2 g (Group H), generally increasing with the cows’ age. The length of the largest left SMLN ranged from 4.14 cm (Group L) to 17.52 cm (Group H), while that of the largest right SMLN ranged from 3.43 cm (Group L) to 15.26 cm (Group H). The width of the largest left SMLN was between 2.32 cm (Group L) and 10.38 cm (Group H), and the width of the largest right SMLN ranged from 1.90 cm (Group L) to 10.34 cm (Group H).

Table 3 presents summary statistics for BW and SMLN morphological parameters stratified by age group. The BW of Group M was significantly higher than that of Group L, while Group H fell between the two with no statistically significant difference. No significant differences were observed among the three groups in the total number of SMLNs. A significant increasing trend was observed with advancing age on left, right, and bilateral SMLN weights. Group L was significantly lower than Group H, while Group M was intermediate with no significant difference from either group. For largest left and right SMLN lengths, Groups L and M were comparable, whereas Group H was significantly greater than Group L; for certain parameters, Group H was also significantly greater than Group M. For largest left and right SMLN widths, Group L was significantly smaller than Group H, while Group M showed no significant difference from either Group L or Group H. Overall, both the weight and dimensions of SMLNs increased significantly with age.

**Figure 2:**
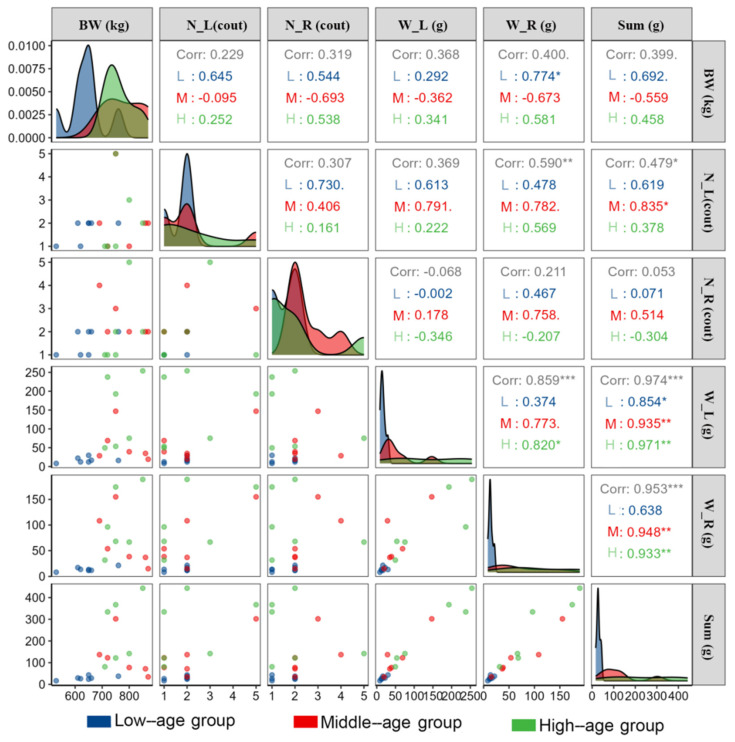
Correlation analysis of main SMLN factors across different age groups of dairy cows. Data points are color-coded by age group: blue = low-age group (L, 2 years, *n* = 7); red = middle-age group (M, 3–5 years, *n* = 6); green = high-age group (H, ≥6 years, *n* = 6). The six variables displayed are: body weight (BW, kg); number of left SMLNs (N_L); number of right SMLNs (N_R); weight of left SMLNs (W_L, g); weight of right SMLNs (W_R, g); and total bilateral SMLN weight (Sum, g). The matrix is read as follows: (1) Diagonal panels show kernel density estimation (KDE) curves for each variable within each age group; wider, flatter curves indicate greater spread, while taller, narrower curves indicate values concentrated around the peak, allowing rapid visual assessment of data distribution and approximate normality within each group. (2) Off-diagonal scatter plots (lower triangle) display pairwise relationships between variables, with each point representing one individual cow, color-coded by age group; clustering or directional trends indicate potential associations. (3) Upper triangle panels report Pearson correlation coefficients: “Corr” gives the overall coefficient across all 19 cows regardless of age group, while r(L), r(M), and r(H) give age-group-specific coefficients for the low-, middle-, and high-age groups, respectively. Significance levels are denoted by asterisks: * *p* < 0.05; ** *p* < 0.01; *** *p* < 0.001. Panels without asterisks indicate non-significant correlations (*p* ≥ 0.05). SMLN—supramammary lymph node; BW—body weight.

Figure 2 presents a correlation analysis of SMLN with 6 factors across different age groups of dairy cows. Regarding the influence of BW on SMLN characteristics: in the low-age group, BW was significantly and positively correlated with right SMLN weight (W_R) (r(L) = 0.774, *p* < 0.05), indicating that heavier young cows tended to have heavier right SMLNs. The correlation between BW and left SMLN weight (W_L) did not reach statistical significance in any age group or in the overall cohort (*p* ≥ 0.05). This finding should be interpreted cautiously, as the corresponding association with left SMLN weight was not statistically significant in this small subgroup. With the current sample size (*n* = 19), the study is underpowered for detecting moderate effect sizes in subgroup analyses. Specifically, the per-group sample sizes of *n* = 6–7 provide approximately 50–60% power to detect a medium-to-large effect (Cohen’s d ≈ 0.8) at α = 0.05 in pairwise comparisons. Accordingly, the BW-SMLN correlation findings and subgroup-specific results should be interpreted as preliminary and hypothesis-generating rather than definitive. Within the high-aged group, right SMLN weight (W_R) is strongly positively correlated with left SMLN weight (W_L) (r (H) = 0.820, *p* < 0.05), suggesting that cows with heavier left SMLNs also have heavier right SMLNs (Figure 2). Across all cows regardless of age group, W_L and W_R were strongly correlated (r = 0.859, *p* < 0.001), highlighting a consistent relationship between the weights of left and right SMLNs.

The results of the post hoc power analysis are summarized in Table 4. For the primary one-way ANOVA comparing bilateral SMLN weight across the three age groups, the observed effect size was large (Cohen’s f = 0.72), yielding an achieved power of approximately 0.78 (78%) at α = 0.05 with the current sample configuration (n_L = 6, n_M = 6, n_H = 7). This value approached but did not reach the conventional threshold of 0.80. For pairwise post hoc comparisons conducted via Tukey’s HSD, the per-group sample size of *n* = 6–7 provided approximately 55–65% power to detect a medium-sized effect (Cohen’s d ≈ 0.8) following family-wise error correction, suggesting that certain biologically meaningful pairwise differences—particularly between Groups M and H—may not have been captured due to insufficient statistical power. Within-group Pearson correlation analyses revealed that the minimum detectable correlation coefficient at 80% power (α = 0.05, two-tailed) was r ≈ 0.76, indicating that moderate correlations (r < 0.76) within individual subgroups may have remained undetected.

## 4. Discussion

The findings of this study highlight the notable individual differences in the morphology, including number, weight, length, and width (Table 2 and Figure 1). Specifically, the length and width of SMLNs observed here were markedly larger than those reported by Khoramian et al. [10], who employed a portable ultrasound with a 2–5 MHz convex transducer. This discrepancy may be attributed to differences in the cow populations examined or limitations in the accuracy of ultrasound measurement techniques. Given the critical role of SMLNs in immune response, such variations could have important implications for the diagnosis and management of udder health, particularly in the context of mastitis and other mammary gland infections. From an anatomical standpoint, the bovine mammary gland’s lymphatic drainage system is architecturally suited to concentrate immune surveillance at the SMLN level. Pandey et al. [2] described that the lymph channels of the bovine udder are as extensive as its venous system and run in parallel, with afferent lymphatic ducts from all four quarters ultimately draining into the supramammary lymph gland. This anatomical arrangement means that even localized intramammary infections generate systemic lymph node responses, which may contribute to the marked weight variability observed in the present study. While the bovine and human mammary systems differ substantially, both share the principle that the lymphoid apparatus adjacent to the gland reflects the immunological history of the tissue. Additionally, the results of this study are consistent with those of Bradley et al. [11], demonstrating that the weight of SMLNs increases with age. Correlation analysis further indicates a positive association between BW and SMLN weight, with a strong concordance between the weights of left and right SMLNs. These findings suggest that SMLN weight and dimensions are significantly associated with age and BW, with marked inter-individual variability (Figure 2). To our knowledge, this is the first study to document such wide–ranging inter-individual variability in SMLN weight (16.6–443.2 g), length, and number (1–5 per side) through direct post mortem dissection in Holstein dairy cows. These data fill an important gap in the literature, as previous anatomical descriptions were largely based on ultrasonographic measurements, which may underestimate true node sizes. A key limitation of this study is the retrospective nature of data collection, which precluded the systematic recording of BCS, complete mastitis history, somatic cell count (SCC) trajectories, and vaccination status for all individual cows. BCS may independently influence metabolic status and immune organ morphology [12], and chronic subclinical mastitis is known to stimulate reactive lymphoid hyperplasia [13]. Given the small sample size (*n* = 19), even a small number of animals with undetected immunological histories could disproportionately influence group means and variability estimates. Future prospective studies should mandate the systematic recording of these covariates prior to sample collection to enable more definitive causal inference. Taken together, these results support the study’s hypothesis that SMLN morphological characteristics are associated with age and BW, thereby providing a preliminary quantitative baseline for SMLN diversity in Holstein dairy cows.

The diversity in the number of SMLNs between the left and right sides of the udder further underscores the need for careful consideration when administering immunomodulators or vaccines. As demonstrated by Camussone et al. [14] and Guan et al. [7], SMLN-targeted intervention can enhance immune responses and improve udder health. However, the present findings highlight that such strategies should account for individual differences in number, laterality, and location to optimize therapeutic efficacy. Previous studies have shown that immunomodulators or vaccines administered near the SMLNs can enhance local immune responses or improve udder health [15,16]. The present anatomical data may help refine such approaches by highlighting inter-individual variability in node number, size, and laterality. Supporting this strategy, Ghaemmaghami et al. [8] reported that in Saanen dairy goats, ultrasonographic examination of SMLNs is a safe, non-invasive method for visualizing these structures, and that SMLN dimensions increase with age, consistent with the age-dependent weight increases documented in the present study. Although that study found no significant relationship between SMLN size and subclinical mastitis status, it confirmed that ultrasonographic SMLN assessment can serve as a baseline monitoring tool in herd health programs. The present study extends these findings by providing direct morphometric data from post mortem dissection, which may better inform the anatomical targeting of immunomodulatory interventions.

In addition to their role in immune modulation, SMLNs are also critical in clinical diagnostics. Several cows in this study exhibited markedly enlarged SMLNs compared to age-matched counterparts, such as the 6-year-old cow (ID 413555) with a bilateral SMLN weight of 443.20 g and a second 6-year-old (ID 113062) with a 334.00 g bilateral weight (Table 2). This phenomenon is consistent with reactive lymphoid hyperplasia triggered by chronic intramammary antigenic stimulation. Supporting this interpretation, Korelidou et al. [17] recently reported in dairy goats that swollen SMLNs constitute a distinct and clinically recognizable udder health category, with characteristic skin surface temperature patterns that differ from those of healthy glands and fibrotic tissue [17]. Although the current study focused on cattle and employed post mortem dissection rather than thermography, the parallel observation of swollen SMLNs as a clinically meaningful finding underscores the diagnostic relevance of SMLN enlargement across ruminant species. The marked asymmetry in Cow 415700 (L: 28.7 g, R: 108.30 g) may reflect a history of mastitis or subclinical infection affecting the right mammary gland, consistent with the known role of ipsilateral lymph node reactivity in mastitis. In the absence of complete lifetime health records, the etiology of these enlarged nodes cannot be definitively determined. Possible explanations include: (1) prior subclinical mastitis episodes that triggered chronic antigen stimulation and reactive lymphoid hyperplasia [13]; (2) prior vaccination with mammary-targeted antigens near the SMLN region; or (3) inherent individual anatomical variation. The SMLN region is a common site for palpation and ultrasonographic examination in veterinary practice. Future studies should prospectively record full mastitis histories, SCC, and vaccination status for each cow to allow a proper investigation of these associations. Notably, in sheep and goats, acute clinical mastitis caused by *S. aureus* or *Mannheimia haemolytica* is frequently associated with oedematous and swollen SMLNs, reflecting the direct involvement of these nodes in the acute inflammatory response [18]. Subclinical mastitis, defined as mammary inflammation without clinically detectable signs, similarly stimulates SMLN reactivity at the cellular level even in the absence of gross enlargement [18]. The marked asymmetry in SMLN weight observed in Cow 415700 in the present study (L: 28.7 g vs. R: 108.3 g) is consistent with the ipsilateral reactivity pattern described in ruminant mastitis pathology, where the lymph node draining the affected mammary quarter becomes preferentially enlarged due to local antigen stimulation [8,18]. Nevertheless, the absence of complete health records is recognized as a significant limitation; future studies should prospectively capture full mastitis history (including SCC thresholds), vaccination schedules, and immunological records prior to sample collection.

The results of this study are consistent with previous research on the role of SMLNs in immune function. For example, Xu et al. [15] reported that immunomodulators administered near the SMLNs had therapeutic effects on subclinical mastitis. Furthermore, Tucker et al. [19] found that *Listeria monocytogenes* could infect the cytoplasm of stromal cells in the lymph nodes (such as fibroblastic reticular cells and blood endothelial cells) and induce a type I interferon response. These findings, combined with the results of the present study, emphasize the importance of SMLNs in maintaining udder health and suggest that further research is needed to explore the relationship between SMLN anatomy and immune function. Importantly, histological and histopathological examination of the SMLNs, particularly those with markedly enlarged sizes, would provide critical information on the underlying cellular architecture, degree of lymphoid activation, and potential pathological changes. Such analyses would greatly enhance the interpretive value of the morphometric data reported here and are strongly recommended for future studies.

In this study, 19 Holstein dairy cows were included, all culled due to reproductive failure at the on-farm slaughterhouse. While the sample size is small, this study provides preliminary insights into the diversity of SMLNs and establishes a reference baseline for future investigation. Future studies should incorporate cohorts of at least 60–80 cows per age group, ideally sourced from multiple farms and geographic regions, to achieve adequate statistical power (≥80%) for detecting clinically meaningful differences in SMLN morphology across age, BW, and parity strata.

## 5. Conclusions

In conclusion, this study provides one of the first contemporary direct post mortem morphometric characterizations of SMLN diversity through direct post mortem dissection in Holstein dairy cows, documenting a wide range of inter-individual variability in node number (1–5 per side), bilateral weight (16.60–443.20 g), length, and width. These findings confirm the study’s central hypothesis: SMLN morphological characteristics are significantly associated with age and positively correlated with BW. Notably, asymmetric node numbers were observed between the left and right sides, and outlier weights were identified in certain cows. Such variability has direct implications for veterinary practice, particularly with respect to diagnostics, vaccine and immunomodulator administration, and early detection of udder health disorders. Future research should incorporate larger, multi-farm cohorts, comprehensive mastitis histories, SCC, and histopathological examination of enlarged SMLNs to further elucidate the immunological significance of the morphological diversity documented here. Furthermore, future prospective studies with larger sample sizes and complete covariate recording should employ ANCOVA or mixed-effects models to formally disentangle the contributions of age, BW, parity, and lactation stage to SMLN morphology.

## Figures and Tables

**Table 1:** The basic information of the 19 slaughtered Holstein dairy cows ^‡^.

Cow ID	Age (Years)	Body Weight (kg)	Parity	Culling Reason	Lactation Stage ^§^	Recent Daily Milk Yield (kg)
417704	2	530	—	Open/Not pregnant	—	—
417538	2	620	—	Repeated insemination failure	—	—
417600	2	650	—	Open/Not pregnant	—	—
117042	2	650	—	Open/Not pregnant	—	—
417528	2	760	—	Open/Not pregnant	—	—
417516	2	660	—	Open/Not pregnant	—	—
417526	2	610	—	Open/Not pregnant	—	—
416546	3	720	2nd	Open/Not pregnant	Peak/mid	26
416531	3	870	—	Repeated insemination failure	—	—
415700	4	690	2nd	Abortion	Extended	25
414645	5	750	3rd	Open/Not pregnant	Peak/mid	30
414839	5	800	3rd	Repeated insemination failure	Peak/mid	30
414630	5	860	3rd	Repeated insemination failure	Peak/mid	32
113062	6	720	4th	Open/Not pregnant	Late	30
413580	6	750	4th	Open/Not pregnant	Peak/mid	31
413555	6	850	4th	Abortion	Peak/mid	32
412689	7	710	5th	Open/Not pregnant	Peak/mid	27
111215	8	800	6th	Open/Not pregnant	Peak/mid	25
410624	9	750	7th	Open/Not pregnant	Peak/mid	22

^‡^ Cows in this study were heterogeneous with respect to parity, lactation stage, and culling reason, which may represent confounding factors. These variables are included in the table for transparency; readers should interpret inter-individual comparisons with this heterogeneity in mind. ^§^ Lactation stage was classified according to the International Dairy Federation (IDF) framework: early lactation—days in milk (DIM) 1–100; peak/mid-lactation—DIM 101–200; late lactation—DIM 201–305; extended lactation—DIM > 305. SMLNs—supramammary lymph nodes. —: not applicable.

**Table 2:** The number, weight (g), length (cm) and width (cm) of SMLNs isolated from left or right side of the udder in 19 slaughtered Holstein dairy cows ^‡^.

Cow ID	Number			Weight (g)			Length (cm) ^#^		Width (cm) ^#^	
	Left	Right	Sum	Left	Right	Sum	Left	Right	Left	Right
417704	1	1	2	8.30	8.30	16.60	4.57	3.61	2.32	2.49
417538	1	1	2	12.40	13.80	26.20	6.07	6.67	2.86	2.58
417600	2	1	3	29.80	13.60	43.40	5.24	3.87	3.59	3.97
117042	2	2	4	12.20	11.70	23.90	9.86	4.31	2.94	2.17
417528	2	2	4	16.20	21.40	37.60	4.14	5.64	3.47	3.67
417516	2	2	4	16.60	12.00	28.60	4.42	4.63	2.89	1.90
417526	2	2	4	22.20	17.30	39.50	5.33	3.43	3.21	2.60
416546	1	2	3	68.90	53.70	122.60	7.80	7.95	5.65	4.64
416531	2	2	4	19.40	15.20	34.60	6.40	5.14	3.08	3.95
415700	2	4	6	28.70	108.30	137.00	4.31	6.04	3.59	5.99
414645	5	3	8	146.90	155.00	301.90	9.29	10.53	5.12	6.81
414839	1	2	3	39.30	38.30	77.60	6.78	8.19	5.79	4.42
414630	2	2	4	34.90	36.80	71.70	4.57	5.97	3.73	3.94
113062	1	1	2	237.70	96.30	334.00	15.09	15.26	8.70	6.43
413580	1	2	3	53.60	68.10	121.70	10.46	8.60	4.34	5.05
413555	2	2	4	254.10	189.10	443.20	17.52	14.93	10.38	10.34
412689	1	1	2	49.60	31.50	81.10	8.44	7.54	6.79	6.99
111215	3	5	8	75.20	66.70	141.90	8.03	7.55	4.58	4.30
410624	5	1	6	192.80	174.30	367.10	9.08	8.68	6.43	7.88

^‡^ Cows in this study were heterogeneous with respect to parity, lactation stage, and culling reason, which may represent confounding factors. These variables are included in the Table 1 for transparency; readers should interpret inter-individual comparisons with this heterogeneity in mind. ^#^ When multiple SMLNs are present, the measurements are derived from the largest SMLN.

**Table 3:** Morphological characteristics of supramammary lymph nodes by age group in slaughtered Holstein dairy cows (mean ± SD).

Parameters	Group L (2 yr, *n* = 7)	Group M (3–5 yr, *n* = 6)	Group H (≥6 yr, *n* = 6)
Body weight (kg)	640.00 ± 68.80 ^a^	781.67 ± 74.14 ^b^	763.33 ± 52.79 ^ab^
Total number of SMLNs	3.29 ± 0.95	4.67 ± 1.97	4.00 ± 2.61
Left SMLN weight (g)	16.81 ± 7.20 ^a^	56.35 ± 47.41 ^ab^	143.83 ± 94.97 ^b^
Right SMLN weight (g)	14.01 ± 4.24 ^a^	67.88 ± 52.99 ^ab^	104.33 ± 63.53 ^b^
Bilateral SMLN weight (g)	30.83 ± 9.63 ^a^	124.23 ± 94.55 ^ab^	248.17 ± 151.49 ^b^
Largest left SMLN length (cm)	5.66 ± 1.96 ^a^	6.53 ± 1.90 ^a^	11.44 ± 3.94 ^b^
Largest right SMLN length (cm)	4.59 ± 1.18 ^a^	7.30 ± 1.98 ^ab^	10.43 ± 3.65 ^b^
Largest left SMLN width (cm)	3.04 ± 0.43 ^a^	4.49 ± 1.17 ^a^	6.87 ± 2.35 ^b^
Largest right SMLN width (cm)	2.77 ± 0.74 ^a^	4.96 ± 1.18 ^b^	6.83 ± 2.15 ^b^

^a,ab,b^ Within each row, values with different superscript letters differ significantly (*p* < 0.05); values sharing the same superscript letter (or with no superscript) are not significantly different.

**Table 4:** Summary of post hoc statistical power analysis.

Analytical Framework	Effect Size Metric	Observed/Assumed Effect Size	Sample Size	Significance Level (α)	Achieved Power	Interpretation
One-way ANOVA (3 age groups)	Cohen’s *f*	0.72 (large)	*n*_L = 7, *n*_M = 6, *n*_H = 6	0.05	~0.78 (78%)	Approaching but below the 0.80 threshold
Pairwise comparison (Tukey’s HSD)	Cohen’s *d*	~0.80 (medium)	*n* = 6–7 per group	0.05 (family-wise corrected)	~0.55–0.65 (55–65%)	Underpowered; risk of Type II error for M vs. H
Within-group Pearson correlation	Correlation coefficient (*r*)	Minimum detectable: *r* ≈ 0.76	*n* = 6–7 per group	0.05 (two-tailed)	0.80 (target)	Moderate correlations (*r* < 0.76) undetectable

## Data Availability

The data presented in this study are available on request from the corresponding author. (please specify the reason for restriction, e.g., the data are not publicly available due to privacy or ethical restrictions.)

## Abbreviations

The following abbreviations are used in this manuscript:

SMLNs	Supramammary lymph nodes
BW	Body weight
N_L	Number of left SMLNs
N_R	Number of right SMLNs
W_L	Weight of left SMLNs
W_R	Weight of right SMLNs
Sum	Total weight of SMLNs
